# Cancer Survival and Travel Time to Nearest Reference Care Center for 10 Cancer Sites: An Analysis of 21 French Cancer Registries

**DOI:** 10.3390/cancers15051516

**Published:** 2023-02-28

**Authors:** Joséphine Gardy, Sarah Wilson, Anne-Valérie Guizard, Véronique Bouvier, Laure Tron, Ludivine Launay, Arnaud Alves, on behalf of the Francim Group, Guy Launoy, Florence Molinié, Joséphine Bryère, Olivier Dejardin

**Affiliations:** 1 Calvados General Tumor Registry, Centre François Baclesse, 14000 Caen, France; 2 Calvados Digestive Cancer Registry, University Hospital of Caen, 14000 Caen, France; 3 ANTICIPE U1086 INSERM-UCN, University of Caen, 14000 Caen, France; 4 FRANCIM Network (French National Network of Cancer Registries), 31000 Toulouse, France; 5 Epidemiology Research and Evaluation Unit, Department of Research, University Hospital of Caen, 14033 Caen, France

**Keywords:** cancer, outcomes, survival, travel time, geographical accessibility

## Abstract

**Simple Summary:**

In this French population based study, travel time had an effect on cancer survival for ten of the eighteen tested combinations (sex/sites). Lower survival was observed in patients residing farthest from the referral center for half of the included cancer sites. The pattern of the effect of travel time was different according to tumor type, being either linear, reverse U-shape, non-significant, or better for more remote patients.

**Abstract:**

Background: The impact of several non-clinical factors on cancer survival is poorly understood. The aim of this study was to investigate the influence of travel time to the nearest referral center on survival of patients with cancer. Patients and methods: The study used data from the French Network of Cancer Registries that combines all the French population-based cancer registries. For this study, we included the 10 most common solid invasive cancer sites in France between 1 January 2013 and 31 December 2015, representing 160,634 cases. Net survival was measured and estimated using flexible parametric survival models. Flexible excess mortality modelling was performed to investigate the association between travel time to the nearest referral center and patient survival. To allow the most flexible effects, restricted cubic splines were used to investigate the influence of travel times to the nearest cancer center on excess hazard ratio. Results: Among the 1-year and 5-year net survival results, lower survival was observed for patients residing farthest from the referral center for half of the included cancer types. The remoteness gap in survival was estimated to be up to 10% at 5 years for skin melanoma in men and 7% for lung cancer in women. The pattern of the effect of travel time was highly different according to tumor type, being either linear, reverse U-shape, non-significant, or better for more remote patients. For some sites restricted cubic splines of the effect of travel time on excess mortality were observed with a higher excess risk ratio as travel time increased. Conclusions: For numerous cancer sites, our results reveal geographical inequalities, with remote patients experiencing a worse prognosis, aside from the notable exception of prostate cancer. Future studies should evaluate the remoteness gap in more detail with more explanatory factors.

## 1. Introduction

Factors related to the tumor and demographic characteristics are the main predictors of cancer prognosis. However, the influence of numerous non-clinical factors such as socioeconomic environment and health care organization, including hospital characteristics, on cancer survival has been gaining increasing interest.

Health care accessibility is defined by the capacity to achieve the best medical outcome in a timely manner, being mainly driven by two components, social access and geography. The influence of social condition on access to health care has been widely studied. Patients with a low socioeconomic status have a lower probability of participating in screening and early detection, leading to a more advanced cancer stage at clinical presentation, as well as a lower probability of being treated in a high volume hospital, both resulting in a lower cancer survival compared to those with a higher socioeconomic status [1,2,3]. These inequalities have been reported in all industrialized countries. In France, where patients can choose their preferred hospital whatever its location or level of specialization, studies have shown that numerous factors such as patient’s age, sex and distance between the hospital and place of residence have an influence on this choice [4,5]. Socio-economic factors can also intervene, even though in France integral reimbursement of all health-related expenditures is guaranteed from cancer diagnosis. However, leading up to diagnosis, the outstanding amount to be paid by the patient can be substantial if the patient relies only on universal insurance health care coverage. This financial burden may restrict access to certain physicians in the private sector, which in turn could delay diagnosis for patients with less financial resources. Moreover, distance to medical facilities can also present a financial burden for patients due to travel costs.

To date, geographic inequalities have been little documented and studies have been limited to a few cancer sites. There are some published studies on the influence of travel time for patients on survival in Europe. The majority of these studies measured the theoretical travel time to the nearest hospital. Kravdal et al. in 2006 [6] in their study on Norwegian data, showed no significant effect of travel time to hospital on survival. In England, Jones et al. [7] also showed no significant difference, with only the journey to the GP having an influence on survival. For colorectal cancer, Sjöström et al. in a Swedish study in 2020 [8] found no evidence of an association between travel time and colorectal cancer survival. In contrast, Murage in 2016 [9] showed that longer average travel times to cancer services were associated with poorer survival. In their study in 2014 [10], Dejardin et al. showed the same phenomenon on French data. 

Kelly et al. in 2016 [11] reviewed 108 papers on studies of data from global north countries. In summary, 77% of the included studies found an association between poorer health status and distance from the health facilities the patient needs to attend. The authors conclude that a distance effect cannot be excluded, and distance and travel time should be taken into account when configuring the location of health facilities and treatment options for patients.

Since the factors that determine where people live depend on both social and geographical criteria, social and geographic inequalities in health are closely linked. It therefore seems essential to integrate, in the analysis of geographic inequalities in cancer survival, information on the social environment of patients in order to identify the specific effect of travel time on survival of patients with cancer. 

Some of the above papers have used either old data, or data that are not population based, or focused on very few tumor types. In addition, only a few studies have considered net survival [9,10], i.e., net from other causes of death and only related to the cancer. 

In order to study the effect of geographical inequalities on cancer survival, use of data from population-based cancer registries guarantees the completeness of cases in the study areas, unlike hospital series or hospital discharge data. Prior to explaining the mechanisms of geographical inequalities, which requires much medical information specific to each cancer site, the illustration and the comparison of such geographical inequalities across cancer sites using the same methodology is a mandatory prerequisite. To our knowledge, no study has conducted a systematic analysis of the geographical inequalities in cancer survival adjusted on social environment for the 10 most frequent cancers.

The aim of this study was to investigate the influence of travel time to the nearest referral center on net survival of patients with one of the ten most common cancers between 2013 and 2015 in France, using data from French cancer registries.

## 2. Patients and Methods 

### 2.1. Study Design and Patient Population

The study used data from the French Network of Cancer Registries (FRANCIM), which combines all the French population-based cancer registries, between 1 January 2013 and 31 December 2015; it included the 10 commonest solid invasive cancer types in France according to the 3rd edition of the International Classification of Diseases for Oncology (ICD-O-3); the included cancers were: breast (C50), colon-rectum and anal canal (C18–C21), lung (C33–C34), pancreas (C25), prostate (C61), skin melanoma (C44—morphology 87203 to 87803), bladder (C67), head and neck (C01–C06; C09–C14), kidney (C64), and liver (C22). Our study excluded hematological malignancies, male breast cancers (because of the limited sample size, N = 309) and pediatric cancers (aged under 15 years, covered by a national pediatric registry). In total, this represented 160,634 cases during the study period.

The area covered by the registries included in our study represents approximatively 20% of the French population. The French departments covered (fully or partially) by a register and included in this study are the following: Calvados, Charente, Charente-Maritime, Côte-d’Or, Doubs, Finistère, Gironde, Hérault, Isère, Loire-Atlantique, Manche, Nord, Haut-Rhin, Saône-et-Loire, Deux-Sèvres, Somme, Tarn, Vendée, Vienne, Haute-Vienne and Territoire-de-Belfort. The completeness and data quality of the included registries are regularly assessed by the International Agency for Research on Cancer (IARC) and the European Network of Cancer Registries (ENCR). This study was approved by the Commission Nationale Informatique et Libertés (CNIL—n°921057).

#### 2.1.1. Variables

Data collected included sex, year of diagnosis, age, and site of primary tumor. Age at diagnosis was separated into groups: 15–54, 55–64, 65–74, and 75+ years. For all diagnosed cancers, patient addresses were geolocalized using Geographic Information Systems (ArcGIS; Esri, Redlands, CA, USA); the exact geographic position (X, Y coordinates) of the patient address and the residential IRIS (Ilôts Regroupés pour l’Information Statistique) were available. 

#### 2.1.2. Travel Time

For each patient, the travel time in minutes from residential address to the reference cancer care center was calculated. The reference cancer care center was defined by the closest University Hospital (*Centre Hospitalier Universitaire*) or Cancer Control Center (*Centre de Lutte Contre le Cancer)* in terms of travel time. Although not as specialised as the centers of reference, non-reference care centers (local and private hospitals) provide comprehensive care for a patient. These travel times were estimated using a road-network database (Multinet TéléAtlas). Travel speeds, computed in minutes, were estimated according to legal speeds for the different road classes. Travel times were separated into four classes: <30, 30–59, 60–89 and 90+ minutes for descriptive purposes, but used in a continuous form in a parametric survival model. 

#### 2.1.3. Social Deprivation

Social deprivation was measured by the French version of the European Deprivation Index (EDI); the principles and methods for building this are detailed in previous papers [12,13]. Briefly, it is an ecological index that measures relative poverty in small geographic areas, based on information from the European Union Survey of Income and Living Conditions (EU-SILC) and census information. The IRIS is the smallest geographic unit in France defined by the “Institut National de la Statistique et des Etudes Economiques” (INSEE); the French version of the EDI is associated with the residential IRIS available. Due to the suspected association between travel time and geographical distribution of socioeconomic deprivation, this index was systematically integrated to the analyses. 

#### 2.1.4. Outcomes

Survival time was defined as the difference between date of diagnosis and date of last contact for vital status. Follow-up ended on 30 June 2018, i.e., patients alive on that date had their survival time censored. Lost to follow-up accounted for about 3.5% of cases overall. The information on vital status was collected through an active standardized search procedure by the French Network of Cancer Registries, based on requests to the “Répertoire National d’Identification des Personnes Physiques” [RNIPP] and, if necessary, other sources of information (including medical records or birthplace public services). Survival time was defined as the difference between the date of last information and the date of cancer diagnosis. When the date of death was the same as the date of cancer diagnosis, those cases were included in the analyses with a survival time equal to 0.5 days, following standard procedure. 

#### 2.1.5. Statistical Analysis

All analyses were conducted in the framework of net survival. This method assumed that patients would only die of their cancer. Thus, as shown in equation 1, the observed mortality for one patient is the sum of the expected mortality and of the mortality in excess (i.e., only related to the cancer):

(1) $$\mu_{observed}=\mu_{expected}+\mu_{excess}$$

Expected mortality could be known either by the registration of cause of death or by the use of a life table to estimate the expected mortality for a comparable patient in terms of age, sex, year of diagnosis, and department. We calculated excess mortality using life tables.

In our study, a flexible parametric survival model [14] was used. Net survival is based on the estimated risk of excess mortality. Net survival probabilities and 95% confidence intervals (CIs) by class of travel time to the nearest referral center were estimated. This method estimates cancer-specific survival and, from these calculated net survivals, the remoteness gap (RGap) was calculated. This corresponds to the difference in net survival between patients living closer to referral centers (between 0 and 30 min) and those living further away (more than 90 min). The 95% CI of the remoteness gap was derived using the variance of the net survival of the patients living closer to referral centers and the variance of the patients living further away. We considered that the difference in net survival between the two groups was statistically significant when the 95% CI of the corresponding remoteness gap did not include zero. Finally, the percentage of variation of net survival was calculated between the two groups. 

To allow the most flexible effects, restricted cubic splines were used to investigate the influence of travel times to the nearest cancer center on excess hazard ratio. These restricted cubic splines were adjusted for year of diagnosis, age, and EDI. Concerning the model selection, the inclusion of age in a survival model is obvious. As all survival analysis produced by FRANCIM were stratified by sex, the same strategy was adopted here [15]. The inclusion of EDI is trickier. Previous publications using FRANCIM data [3,16] have clearly established the association between deprivation and survival for the vast majority of cancer sites. To study the proper effect of travel times, deprivation index was systematically included in all analysis. The choice of the number of nodes for the splines was made using the minimization of Akaike information criteria (AIC). The use of restricted cubic splines allowed us to obtain detailed information on the pattern of the effects of travel time. However, before the first node and after the last node, the splines are linear by definition. The position of each node is represented on the graphs. The interpretation of the curves after the last node should be made with caution. 

Finally, a non-proportional effect of travel time was systematically tested. A significant non-proportional effect denotes that the association between travel time and survival was not constant throughout the natural course of the disease. Cases with missing values for travel time were excluded from these analyses, corresponding to complete cases analysis. This represented 0.82% of the data. Our analyses were all stratified by sex and cancer site.

All statistical analyses were performed using the stmp2, mkpline and xblc packages in STATA SE 16 software (StataCorp LLC, College Station, TX, USA).

## 3. Results

The study included a total of 160,634 cases. Characteristics of the population are presented in Table 1. More than half of the cases in this study were men (57.12%). The median age for men was 68.67 years while for women it was 66.57 years.

Table 2 and Table 3 provide net survival probabilities and 95% confidence intervals in each class of travel time and the remoteness gap (RGap) for each cancer site in men and women, respectively. One- and 5-year net survival were higher in patients living closer to referral centers than patients living further away for all cancer sites except head and neck cancer (statistically significant) and pancreas cancer in men, and except head and neck cancer in women. In men, the remoteness gap in relation to 1- and 5-year net survival was in favor of better survival for those living closest to referral centers and statistically significant for lung cancer (RGap_1-year_ = 3.75 [0.82;6.69]; RGap_5-year_ = 3.26 [0.85;5.66]) and skin melanoma (RGap_1-year_ = 3.54 [0.69;6.38]; RGap_5-year_ = 9.82 [2.66;16.99]). In women, the remoteness gap regarding 1-year net survival was statistically significant for lung cancer (RGap_1-year_ = 7.31 [2.80;11.82]) and pancreas cancer (RGap_1-year_ = 7.97 [2.32;13.62]). At 5 years, the remoteness gap was statistically significant for lung cancer (RGap_5-year_ = 7.27 [3.27;11.27]), pancreas cancer (Rgap_5-year_ = 4.47 [1.78;7.15]), and skin melanoma (RGap_5-year_ = 6.50 [0.14;12.87]). The tables also show the variation in 1- and 5-year net survival between patients living closer to referral centers and patients living further away for each cancer site in men and women. The largest decline in 1- and 5-year net survival was observed for lung cancer in men (Δ = 7.83% and 16.45%, respectively) and for pancreas cancer in women (Δ = 20.54% and 41.97%, respectively). 

The restricted cubic splines of the effect of travel time on excess mortality ratio highlighted 4 patterns: 

### 3.1. Linear Pattern

**Figure 1:**
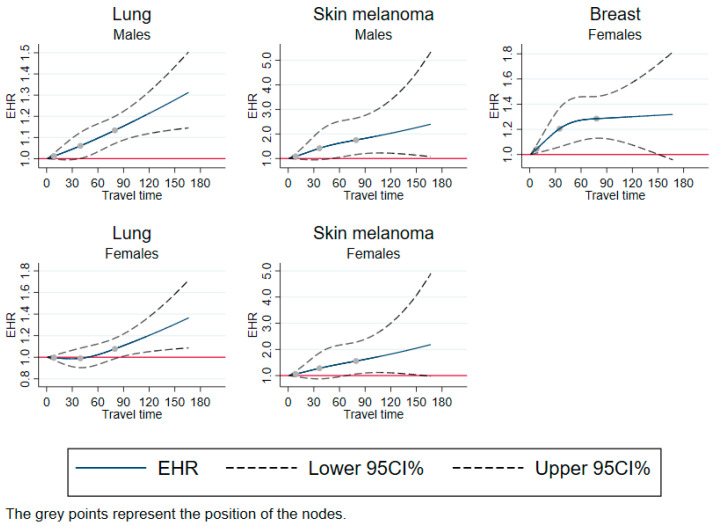
Excess mortality rates as a function of travel time, using restricted cubic splines—cases with linear pattern. EHR: excess hazard ratio; CI: confidence Interval; travel time: travel time in minutes. Adjusted for year of diagnosis, age, and European Deprivation Index.

Figure 1 shows the restricted cubic splines of the effect of travel time on excess mortality for cancer sites where the excess hazard ratio was higher with increasing travel time. This trend was observed for lung cancer and skin melanoma in both sexes, and for breast cancer in women. For lung cancer, the excess hazard ratio reached a maximum of 1.3 in men and 1.35 in women. For skin melanoma, the excess hazard ratio reached a maximum of 2.2 in men and 2.1 in women. For breast cancer in women, the excess hazard ratio reached a maximum of 1.3. 

### 3.2. Reverse U-Shape Pattern

**Figure 2:**
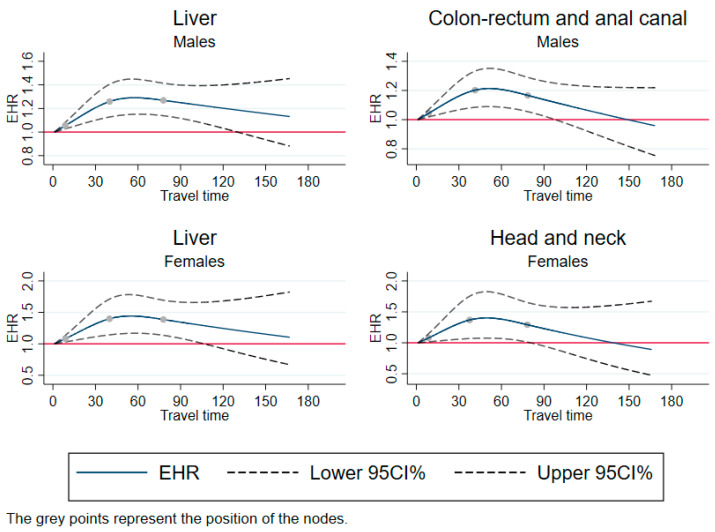
Excess mortality rates as a function of travel time, using restricted cubic splines—cases with reverse U-shape pattern. EHR: excess hazard ratio; CI: confidence Interval; travel time: travel time in minutes Adjusted for year of diagnosis, age, and European Deprivation Index.

Figure 2 shows the restricted cubic splines with reverse u-shape trends. These trends were observed for liver cancer in both sexes, for colon-rectum and anal canal cancer in men, and for head and neck cancer in women. These trends meant that the excess mortality rate increased and then decreased. For liver cancer in men, the excess hazard ratio reached a maximum of 1.3 at 50 min of travel time, and 1.45 at 50 min in women, and then decreased. For colon-rectum and anal canal cancer in men, the excess hazard ratio reached a maximum of 1.2 at 50 min of travel time. For head and neck cancer in women, the excess hazard ratio reached a maximum of 1.4 at 50 min of travel time. 

### 3.3. No Association

**Figure 3:**
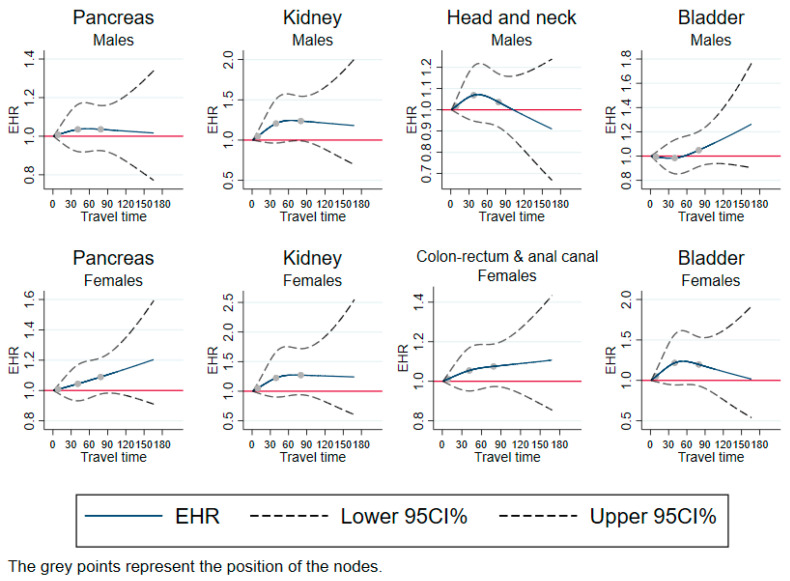
Excess mortality rates as a function of travel time, using restricted cubic splines—cases with no association. EHR: excess hazard ratio; CI: confidence Interval; travel time: travel time in minutes. Adjusted for year of diagnosis, age, and European Deprivation Index.

Figure 3 shows the restricted cubic splines for cancer types with no significant results: pancreas cancer, kidney cancer, and bladder cancer in both sexes; head and neck cancer in men; and colon-rectum and anal canal cancer in women. 

### 3.4. Better Prognosis from Remote Patients

**Figure 4:**
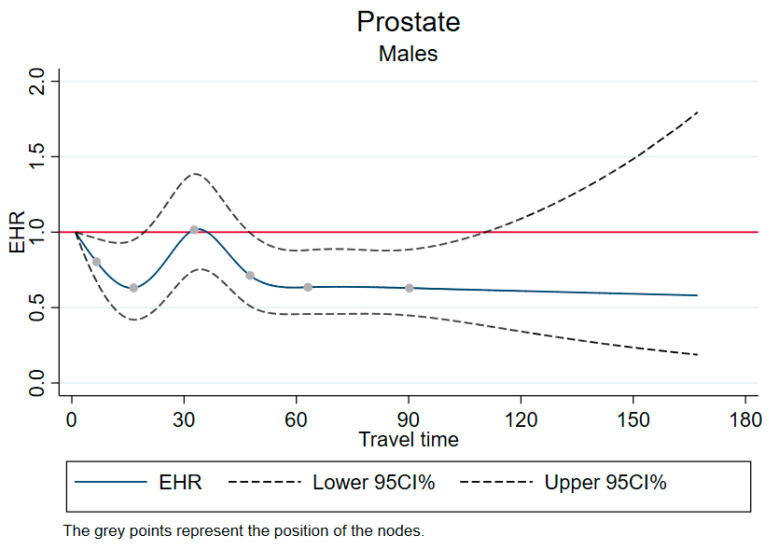
Excess mortality rates as a function of travel time, using restricted cubic splines—cases with better prognosis for remote patients. EHR: excess hazard ratio; CI: confidence Interval; travel time: travel time in minutes. Adjusted for year of diagnosis, age, and European Deprivation Index.

Figure 4 shows the restricted cubic splines for prostate cancer, which were associated with a better prognosis for remote patients (excess hazard ratio was lower for patients living further from referral centers).

For all patterns of excess mortality, the corresponding net survival curves are available in supplementary material (Figures S1–S4). 

## 4. Discussion

Through flexible modelling of the effect of travel time to care center on excess mortality, our study shows that half of cancer types studied were subject to geographical inequalities in survival (4/9 cancer sites in men and 5/9 cancer sites in women demonstrating linear or reverse U-shape patterns, and 1 type with a better prognosis in patients living in the most remote areas). The remoteness gap in net survival for the linear pattern was estimated to be almost 10% at 5 years for skin melanoma in men and 7% for lung cancer in women. This geographical gap in survival is directly linked to cancer-specific mortality and not the consequence of other causes of death. This remoteness gap constitutes a real loss of opportunity for these remote patients and should be considered in future studies and clinical practice. 

An interesting finding was that the pattern of the effect of travel time is highly different by cancer type, being either linear, reverse U-shape, non-significant, or better for more remote patients. The explanation of such patterns cannot be addressed in a low-resolution study like this. On one hand, the centralization of care for some tumor types may play a role in the linear pattern, and the opposite may be true for the reverse U-shape patterns. The mechanism of such geographical inequalities should be studied in more detailed population-based studies according to tumor types. 

A potential explication for the worse survival for patients living further out could be that those patients have a greater delay between symptom onset and cancer diagnosis. The influence of diagnostic delay has been highlighted in numerous publications [17,18]. Unfortunately, date of first symptom is very hard to collect even in dedicated surveys [19] and, thus, is not collected in cancer registries. Moreover, previous publications in France [4,20,21] or elsewhere [22], have provided evidence that patients living further from referral centers are mainly treated in local hospitals. This preference for proximity could also explain in part our results.

Using comparable data (all cancer registries in France) and comparable statistical modelling (flexible net survival models), Tron et al. published a systematic study on the association between socioeconomic environment and excess mortality. Deprivation gaps were calculated between the most deprived and the least deprived. When compared to our remoteness gap, we found similarities for pancreas cancer at 1 year in women (Deprivation Gap = 7.2 [1.6;12.8] vs Remoteness Gap = 7.97 [2.32;13.62]) and for lung cancer at 5 years in men (Deprivation Gap = 2.9 [0.7;5.0] vs Remoteness Gap = 3.26 [0.85;5.66]). Our study reveals that the magnitude and direction of both effects would be similar for some cancer types. Kelly et al. [11] highlighted 3 different patterns for the effect of travel time on survival. Firstly, “distance decay association”, which is an association between patients living closer to a health facility and having better health outcomes/access to health services, compared to those living farther away. Thanks to our flexible modelling of travel time, we can highlight the heterogeneity according to cancer types inside the “distance decay association” group. Secondly, “distance bias association” which, in contrast to the first pattern, is an association between patients living farther away from the health care facility and having better health outcomes/access rates to health services compared to those living closer. Prostate cancer is the only cancer type in which this pattern was detected in our study. The third pattern found in this review is the absence of association between travel time and survival. We confirmed this absence of association for numerous cancer sites using a large study population and flexible statistical models.

An early study in Scotland showed strong evidence that increasing distance from a cancer center is associated with worse survival. A subsequent study in England showed that longer average travel times are associated with worse survival [9]. Illustrating a distance bias association, another study in Scotland from the same group showed that patients living in a rural area and travelling farther to a GP have a lower likelihood of emergency admissions and a better survival [23]. Other studies have examined the effect of living in rural or urban areas on survival, which is another way to attempt to understand geographical inequalities. A study in Denmark in 2018 [24] showed that there was a better survival rate for pancreatic cancer patients for those living in urban areas compared to those living in rural areas. Two systematic literature reviews in 2018 and 2019 have also shown this association for cancer patients. Carriere et al. [25], in their international review and meta-analysis, report that there is strong evidence of an association between rural residence and poor cancer survival outcomes. Afshar et al. [26] conducted a systematic review of studies from Australia, the USA, Europe, Canada and New Zealand. In this review they showed that cancer patients living in rural and remote areas had poorer survival than those living in urban areas.

Our study has a number of strengths. First, this study is based on data from French cancer registries, which guarantee high quality data and the exhaustiveness of all cases in the studied area, and thus a high statistical power. For survival analyses, in our study we used excess base rate modelling from Royston models [27], unlike most other studies that use the Cox model, and we used the maximum likelihood facilities to fit our models without the unrealistic clinical hypotheses required by the Cox model. Moreover, thanks to the use of flexible modelling of the effect of distance, our study shows that the pattern of the effect of travel time is notably different according to cancer type. Four patterns of association were identified in our study: linear pattern, reverse U-Shape pattern, no association, and better prognosis for more isolated patients. To our knowledge, only one study limited to colorectal cancer used a comparable methodology (net survival and spline modelling for travel times). Dejardin et al. showed that the effect of travel-time to the nearest reference cancer center for colorectal cancer patients was a reverse U-shape in France and not significant in England [10]. This previous study highlights the impact of individuality of health care systems, with no association found for England, potentially due to GP gate keeping, and with an association found in France, potentially due to free hospital choice. Our study confirms this influence of geographical accessibility on colorectal cancer on a bigger population, with a similar reverse U-shape found for colorectal cancer. 

Our study has some limitations. First, there was a lack of data about stage at diagnosis and the actual care facility attended by the patients. At a national scale, detailed data on stage and hospital facility attended were not available in an exhaustive way in all French cancer registries. However, the aim of this study was to describe the effect of travel-time to the nearest cancer center and survival using the most extensive data available in France. It is therefore necessary to first establish the dynamics of cancer survival according to travel time to cancer centres, then to add potential mediating factors to explain these dynamics. Cancer stage at diagnosis appears essential to help explain the associations found. Information on the actual center attended by patients would also be important to check that there is no underestimation of travel times for some patients who may travel further than to the nearest center. Stage and place of treatment, which are important to account for, will be analyzed in a future study to explain the different patterns of remoteness gap in survival highlighted in our study. Another limitation was the use of travel time to the nearest cancer center. As aforementioned, place of treatment was not known in this study. Most European studies calculated travel time from the patient’s home (home postal code or specific address) to the nearest cancer referral center or hospital [6,7,8,9,10]. However, as mentioned by Kelly et al. in their review, the use of the nearest referral center is only a proxy of geographic isolation. Due to the variety of public transport available depending on the area, alongside the modalities such as timetables, etc., the integration of these parameters in a GIS would have been extremely complicated. Thus, we made the assumption that road car travel-time is a good proxy for accessibility [28]. Recently indexes of isolation such as APL [29] or Scale [30] have been developed to capture the heterogeneity of spatial accessibility. Nonetheless, such indexes mainly refer to spatial access to primary health care rather than access to specialized cancer centers. Depending on health care system organization, previous publications have shown that both components of spatial accessibility could be associated with cancer prognosis. In the UK, access to primary health care seems more critical rather than access to reference cancer centers in contrast to France [7,10]. Another limitation of this study is the representativeness of the data. We have a large population but this represents only a little more than 20% of the French population, and there are no large French cities in the areas covered by cancer registries. Finally, concerning statistical analysis, we estimated survival using additive net survival models and general population life tables. Under the hypothesis of a geographical gradient in background mortality, this implies that we might have overestimated the gradient because geographical life tables do not exist. This problem also arises with studies on social inequalities that use the EDI. However, a previous publication highlights that, even if causes of death are available, the use of a life table is recommended [31].

## 5. Conclusions

Our results suggest that, for a non-negligible number of cancer types, the travel time by road between a patient’s home and the nearest referral center has an effect on net survival. This geographical gap in survival is directly linked to cancer-specific mortality and not the consequence of other causes of death, and the pattern of the effect of remoteness on cancer survival varies among cancer sites. Geographical inequalities have been previously underestimated relative to social inequalities. However, our study shows that the magnitude of both effects could be comparable. From a clinical and public health perspective, French authorities decided in 2016 to encourage reference hospitals to share medical expertise with local hospitals to provide better medical support for remote patients (*Groupe Hospitalier Territorial*). Further studies in the coming years should evaluate the effectiveness of this strategy. 

## Figures and Tables

**Table 1:** Characteristics of population.

Travel Time in Min	[0;30]				[30;60]				[60;90]				[90;++]				Missing				Total			
	*n* = 63,636				*n* = 55,850				*n* = 32,586				*n* = 7248				*n* = 1314				*n* = 160,634			
Sex	Male		Female		Male		Female		Male		Female		Male		Female		Male		Female		Male		Female	
	*n* = 34,741	%	*n* = 28,895	%	*n* = 32,866	%	*n* = 22,984	%	*n* = 18,984	%	*n* = 13,602	%	*n* = 4356	%	*n* = 2892	%	*n* = 810	%	*n* = 504	%	*n* = 91,757	%	*n* = 68,877	%
Median age (year)	68		66		69		67		69		68		70		68		71		71		69		67	
Age in classes																								
[15;55]	3942	11.35	7516	26.01	3218	9.79	5415	23.56	1662	8.75	2992	22.00	345	7.92	536	18.53	75	9.26	102	20.24	9242	10.07	16,561	24.04
[55;65]	9251	26.63	6448	22.32	8415	25.60	4894	21.29	4706	24.79	2883	21.20	1002	23.00	656	22.68	168	20.74	91	18.06	23,542	25.66	14,972	21.74
[65;75]	11,236	32.34	6464	22.37	11,152	33.93	5235	22.78	6488	34.18	3130	23.01	1553	35.65	703	24.31	243	30.00	94	18.65	30,672	33.43	15,626	22.69
[75;++]	10,312	29.68	8467	29.30	10,081	30.67	7440	32.37	6128	32.28	4597	33.80	1456	33.43	997	34.47	324	40.00	217	43.06	28,301	30.84	21,718	31.53
EDI’s quintile																								
Q1	9597	27.62	7788	26.95	4287	13.04	2917	12.69	2327	12.26	1634	12.01	345	7.92	215	7.43	1	0.12	2	0.40	16,557	18.04	12,556	18.23
Q2	6387	18.38	5235	18.12	6672	20.30	4480	19.49	4344	22.88	2916	21.44	969	22.25	598	20.68	1	0.12	0	0.00	18,373	20.02	13,229	19.21
Q3	5051	14.54	4443	15.38	7606	23.14	5229	22.75	4588	24.17	3253	23.92	1324	30.39	932	32.23	2	0.25	1	0.20	18,571	20.24	13,858	20.12
Q4	5410	15.57	4708	16.29	7620	23.19	5318	23.14	4515	23.78	3315	24.37	1202	27.59	762	26.35	1	0.12	0	0.00	18,748	20.43	14,103	20.48
Q5	7379	21.24	6074	21.02	6137	18.67	4637	20.17	3167	16.68	2466	18.13	515	11.82	385	13.31	0	0.00	1	0.20	17,198	18.74	13,563	19.69
Missing	917	2.64	647	2.24	544	1.66	403	1.75	43	0.23	18	0.13	1	0.02	0	0.00	805	99.38	500	99.21	2310	2.52	1568	2.28
Year of diagnosis																								
2013	11,306	32.54	9451	32.71	10,764	32.75	7456	32.44	6511	34.30	4583	33.69	1381	31.70	972	33.61	297	36.67	180	35.71	30,259	32.98	22,642	32.87
2014	11,445	32.94	9569	33.12	11,025	33.55	7978	34.71	6493	34.20	4748	34.91	1487	34.14	953	32.95	233	28.77	142	28.17	30,683	33.44	23,390	33.96
2015	11,990	34.51	9875	34.18	11,077	33.70	7550	32.85	5980	31.50	4271	31.40	1488	34.16	967	33.44	280	34.57	182	36.11	30,815	33.58	22,845	33.17
Solid tumor sites ^1^																								
Bladder	2218	6.38	539	1.87	2177	6.62	468	2.04	1208	6.36	265	1.95	283	6.50	66	2.28	50	6.17	16	3.17	5936	6.47	1354	1.97
Breast			14,864	51.44			10,973	47.74			6311	46.40			1367	47.27			138	27.38			33,653	48.86
Colon-rectum	5711	16.44	5247	18.16	5715	17.39	4769	20.75	3617	19.05	2929	21.53	713	16.37	543	18.78	80	9.88	97	19.25	15,836	17.26	13,585	19.72
Head and neck	2202	6.34	683	2.36	1963	5.97	542	2.36	1012	5.33	306	2.25	225	5.17	69	2.39	31	3.83	16	3.17	5433	5.92	1616	2.35
Kidney	1758	5.06	855	2.96	1580	4.81	748	3.25	905	4.77	454	3.34	201	4.61	116	4.01	61	7.53	34	6.75	4505	4.91	2207	3.20
Liver	2088	6.01	546	1.89	1933	5.88	446	1.94	1143	6.02	296	2.18	245	5.62	53	1.83	58	7.16	18	3.57	5467	5.96	1359	1.97
Lung	6571	18.91	2965	10.26	6108	18.58	2360	10.27	3567	18.79	1424	10.47	847	19.44	334	11.55	134	16.54	56	11.11	17,227	18.77	7139	10.36
Pancreas	1585	4.56	1546	5.35	1418	4.31	1345	5.85	925	4.87	891	6.55	185	4.25	175	6.05	42	5.19	41	8.13	4155	4.53	3998	5.80
Prostate	11,004	31.67			10,701	32.56			5889	31.02			1464	33.61			277	34.20			29,335	31.97		
Skin melanoma	1604	4.62	1650	5.71	1271	3.87	1333	5.80	718	3.78	726	5.34	193	4.43	169	5.84	77	9.51	88	17.46	3863	4.21	3966	5.76

^1^ Except hematological malignancies.

**Table 2:** One and five-year net survival probabilities and 95% confidence intervals and remoteness gap (RGap), by cancer site, in men.

	1-Year Net Survival					
	[0;30]	[30;60]	[60;90]	[90;++]	Remoteness Gap [95% CI]	Percentage of Variation at 1 Year
Solid Tumor Sites ^1^						
Bladder (C67; all morphology)	77.12 (75.56–78.70)	76.66 (75.09–78.26)	75.94 (73.90–78.04)	74.54 (70.51–78.79)	2.58 (−1.73;6.89)	−3.35
Colon-rectum (C18-C21; all morphology)	83.79 (82.92–84.66)	82.01 (81.11–82.93)	81.59 (80.49–82.71)	82.55 (80.28–84.87)	1.24 (−1.18;3.67)	−1.48
Head and neck (C01-C06,C09-C14; all morphology)	72.24 (70.70–73.81)	71.76 (70.15–73.41)	70.78 (68.61–73.02)	76.82 (72.82–81.04)	**−4.58 (−8.86;−0.29)**	6.34
Kidney (C64; all morphology)	88.19 (86.79–89.61)	85.50 (83.90–87.12)	83.97 (81.85–86.15)	85.85 (81.74–90.16)	2.34 (-1.99;6.67)	−2.65
Liver (C22; all morphology)	50.88 (49.00–52.82)	46.72 (44.79–48.74)	44.73 (42.30–47.30)	49.15 (44.14–54.73)	1.72 (−3.63;7.07)	−3.40
Lung (C33-C34; all morphology)	47.91 (46.86–48.99)	45.76 (44.68–46.87)	43.84 (42.46–45.26)	44.16 (41.42–47.08)	**3.75 (0.82;6.69)**	−7.83
Pancreas (C25; all morphology)	37.59 (35.50–39.81)	35.62 (33.46–37.92)	35.83 (33.21–38.66)	40.44 (34.95–46.79)	−2.85 (−8.72;3.03)	7.58
Prostate (C61; all morphology)	98.11 (97.83–98.39)	97.67 (97.35–97.99)	98.20 (97.85–98.55)	98.07 (97.39–98.75)	0.04 (−0.69;0.77)	−0.04
Skin melanoma (C44; 87203 to 87803)	96.81 (96.00–97.62)	95.31 (94.24–96.38)	95.09 (93.73–96.46)	93.27 (90.54–96.08)	**3.54 (0.69;6.38)**	−3.66
	**5-year Net Survival**					
	**[0;30** **]**	**[30;60** **]**	**[60;90** **]**	**[90;++** **]**	**Remoteness Gap [95% CI]**	**Percentage of Variation at 5 years**
Solid tumor sites ^1^						
Bladder (C67; all morphology)	51.82 (49.31–54.46)	51.04 (48.54–53.67)	49.84 (46.59–53.32)	47.55 (41.34–54.68)	4.27 (−2.42;10.97)	−8.24
Colon-rectum (C18–C21; all morphology)	62.69 (61.12–64.31)	59.25 (57.66–60.88)	58.45 (56.49–60.47)	60.27 (56.05–64.80)	2.42 (−2.07;6.92)	−3.86
Head and neck (C01–C06,C09–C14; all morphology)	39.69 (37.45–42.06)	38.94 (36.62–41.41)	37.45 (34.34–40.83)	47.26 (40.65–54.94)	**−7.57 (−14.55;−0.59)**	19.07
Kidney (C64; all morphology)	76.77 (74.33–79.29)	71.93 (69.22–74.75)	69.26 (65.68–73.03)	72.55 (65.47–80.39)	4.22 (−3.26;11.7)	−5.50
Liver (C22; all morphology)	20.32 (18.56–22.25)	16.62 (14.99–18.44)	15.00 (13.10–17.19)	18.74 (14.53–24.16)	1.59 (−2.97;6.15)	−7.78
Lung (C33–C34; all morphology)	19.82 (18.82–20.86)	17.91 (16.94–18.93)	16.29 (15.14–17.53)	16.56 (14.37–19.09)	**3.26 (0.85;5.66)**	−16.45
Pancreas (C25; all morphology)	10.33 (8.92–11.95)	9.11 (7.76–10.69)	9.24 (7.66–11.13)	12.23 (8.68–17.23)	−1.91 (−5.72;1.91)	18.39
Prostate (C61; all morphology)	93.62 (92.80–94.44)	92.18 (91.31–93.06)	93.92 (92.82–95.02)	93.49 (91.32–95.71)	0.13 (−2.19;2.45)	−0.14
Skin melanoma (C44; 87203 to 87803)	90.46 (88.40–92.58)	86.21 (83.59–88.90)	85.59 (82.11–89.22)	80.64 (73.77–88.14)	**9.82 (2.66;16.99)**	−10.86

^1^ Except hematological malignancies.

**Table 3:** One and five-year net survival probabilities and 95% confidence intervals and remoteness gap (RGap), by cancer site, in women.

	1-Year Net Survival					
	[0;30]	[30;60]	[60;90]	[90;++]	Remoteness Gap [95% CI]	Percentage of Variation at 1 Year
Solid tumor sites ^1^						
Bladder (C67; all morphology)	65.36 (61.71–69.23)	61.31 (57.38–65.52)	63.74 (58.70–69.22)	60.83 (51.39–71.99)	4.53 (−5.58;14.65)	−6.93
Breast (C50; all morphology)	97.81 (97.60–98.02)	97.34 (97.08–97.60)	97.29 (96.98–97.60)	97.25 (96.65–97.86)	0.56 (−0.08;1.19)	−0.57
Colon-rectum (C18–C21; all morphology)	82.99 (82.07–83.93)	81.32 (80.32–82.33)	81.06 (79.83–82.30)	81.15 (78.53–83.86)	1.85 (−0.93;4.63)	−2.22
Head and neck (C01–C06,C09–C14; all morphology)	79.20 (76.69–81.79)	75.89 (72.97–78.93)	75.51 (71.80–79.41)	81.20 (74.56–88.42)	−2.00 (−9.09;5.10)	2.53
Kidney (C64; all morphology)	86.94 (84.86–89.08)	85.12 (82.80–87.50)	85.08 (82.14–88.13)	83.41 (77.58–89.68)	3.53 (−2.66;9.72)	−4.06
Liver (C22; all morphology)	47.11 (43.49–51.03)	41.55 (37.70–45.79)	38.42 (33.84–43.62)	45.42 (35.23–58.57)	1.68 (−9.14;12.50)	−3.59
Lung (C33–C34; all morphology)	55.60 (54.03–57.21)	54.69 (52.97–56.46)	54.42 (52.27–56.67)	48.29 (44.06–52.93)	**7.31 (2.80;11.82)**	−13.15
Pancreas (C25; all morphology)	38.80 (36.66–41.05)	34.66 (32.45–37.01)	34.52 (31.89–37.38)	30.83 (25.59–37.13)	**7.97 (2.32;13.62)**	−20.54
Skin melanoma (C44; 87203 to 87803)	96.12 (95.29–96.96)	95.02 (93.96–96.09)	94.35 (92.92–95.81)	93.03 (89.92–96.24)	3.10 (−0.12;6.31)	−3.21
	**5-year Net Survival**					
	**[0;30** **]**	**[30;60** **]**	**[60;90** **]**	**[90;++** **]**	**Remoteness Gap [95% CI]**	**Percentage of Variation at 5 years**
Solid tumor sites ^1^						
Bladder (C67; all morphology)	44.30 (39.70–49.43)	39.20 (34.45–44.59)	42.22 (36.05–49.46)	38.60 (27.90–53.40)	5.70 (−5.95;17.34)	−12.87
Breast (C50; all morphology)	90.46 (89.79–91.13)	88.50 (87.68–89.33)	88.29 (87.21–89.38)	88.14 (85.81–90.55)	2.31 (−0.12;4.74)	−2.56
Colon-rectum (C18–C21; all morphology)	62.27 (60.67–63.92)	59.13 (57.43–60.87)	58.64 (56.53–60.84)	58.81 (54.16–63.86)	3.46 (−1.46;8.38)	−5.56
Head and neck (C01–C06,C09–C14; all morphology)	54.72 (50.65–59.12)	49.01 (44.55–53.91)	48.37 (42.66–54.84)	58.36 (46.94–72.57)	−3.64 (−15.77;8.49)	6.65
Kidney (C64; all morphology)	76.02 (72.61–79.59)	72.93 (69.21–76.86)	72.87 (68.14–77.93)	70.09 (60.90–80.68)	5.93 (−3.88;15.74)	−7.80
Liver (C22; all morphology)	18.04 (14.93–21.79)	13.55 (10.75–17.09)	11.34 (8.41–15.29)	16.60 (9.27–29.73)	1.43 (−6.53;9.40)	−7.98
Lung (C33–C34; all morphology)	26.88 (25.24–28.64)	25.91 (24.13–27.82)	25.63 (23.42–28.05)	19.61 (15.97–24.09)	**7.27 (3.27;11.27)**	−27.05
Pancreas (C25; all morphology)	10.65 (9.18–12.35)	8.15 (6.85–9.70)	8.08 (6.59–9.91)	6.18 (3.93–9.72)	**4.47 (1.78;7.15)**	−41.97
Skin melanoma (C44; 87203 to 87803)	91.48 (89.80–93.19)	89.13 (87.02–91.30)	87.73 (84.86–90.69)	84.97 (78.83–91.60)	**6.50 (0.14;12.87)**	−7.12

^1^ Except hematological malignancies.

## Data Availability

The data that supports the findings of our study are available from the corresponding author upon reasonable request.

## Supplementary Materials

The following supporting information can be downloaded at: https://www.mdpi.com/article/10.3390/cancers15051516/s1, Figure S1: Survival probability—cases with linear pattern; Figure S2: Survival probability—cases with reverse U-shape pattern; Figure S3: Survival probability—cases with no association; Figure S4: Survival probability—cases with better prognosis for remote patients.

## Appendix A

Members of the French Network of Cancer Registries (FRANCIM) included in the study: Arnaud ALVES (Registre des cancers digestifs du Calvados), Simona BARA (Registre des cancers de la Manche), Anne-Marie BOUVIER (Registre Bourguignon des cancers digestifs), Marc COLONNA (Registre général des cancers de l’Isère), Gaëlle COUREAU (Registre général des cancers de la Gironde), Sandrine DABAKUYO YONLI (Registre des cancers du sein et des cancers gynécologiques de Côte d’Or), Tania D’ALMEIDA (Registre général des cancers en région Limousin), Gautier DEFOSSEZ (Registre des cancers de Poitou-Charentes), Pascale GROSCLAUDE (Registre des cancers généraux du Tarn), Anne-Valérie GUIZARD (Registre général des tumeurs du Calvados), Karima HAMMAS (Registre des cancers du Haut-Rhin), Bénédicte LAPOTRE-LEDOUX (Registre général des cancers de la Somme), Florence MOLINIE (Registre des tumeurs de Loire-Atlantique/Vendée), Jean-Baptiste NOUSBAUM (Registre Finistérien des tumeurs digestives), Sandrine PLOUVIER (Registre des cancers de Lille et de sa région), Brigitte TRETARRE (Registre général des tumeurs de l’Hérault), Anne-Sophie WORONOFF (Registre des tumeurs du Doubs et du Territoire de Belfort).
