# Supplementary material for: Cancer Survival and Travel Time to Nearest Reference Care Center for 10 Cancer Sites: An Analysis of 21 French Cancer Registries
Source: Cancers (Basel). 2023 Feb 28;15(5):1516. doi: 10.3390/cancers15051516 (PMC10000621; doi:10.3390/cancers15051516)
Supplement: Supplementary file 1 [file cancers-15-01516-s001.zip › cancers-2234086-supplementary.pdf]

Figure S1 – Survival probability – cases with linear pattern

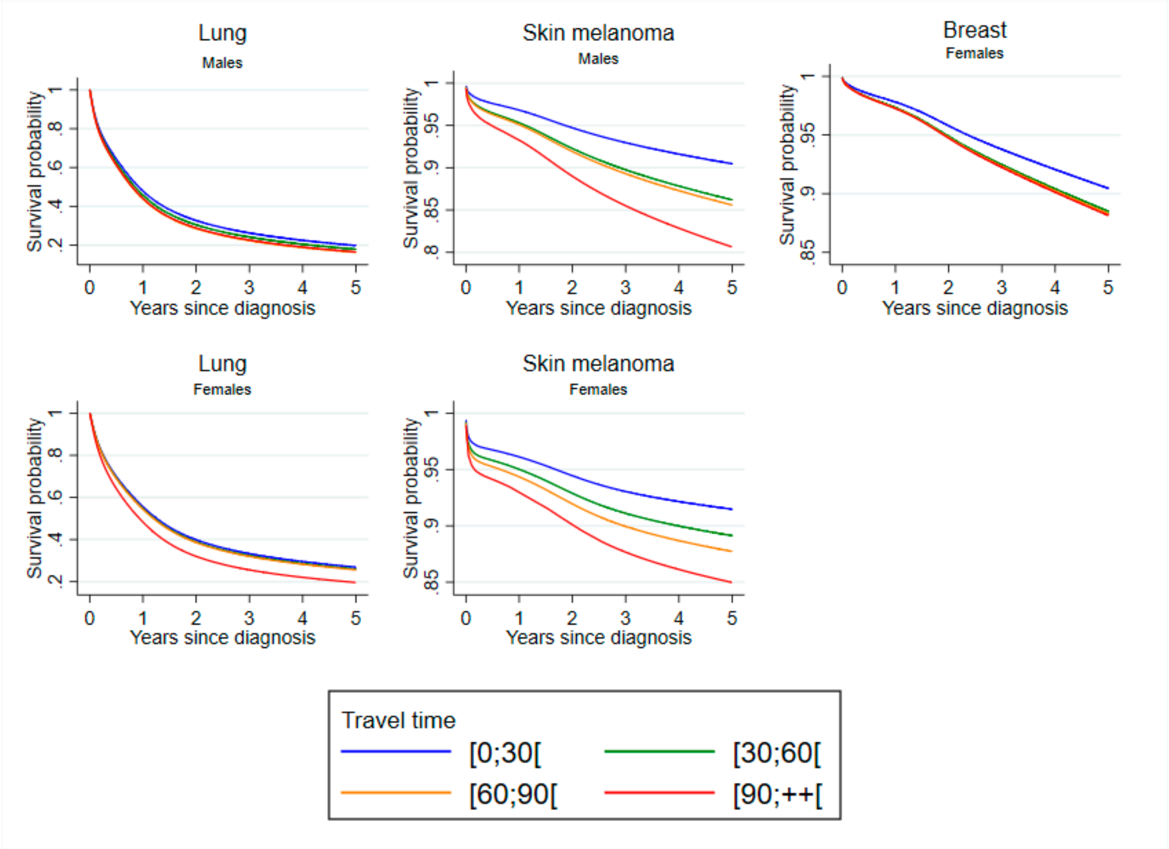

Travel time: travel time in minutes

Figure S2 – Survival probability – cases with reverse U-shape pattern

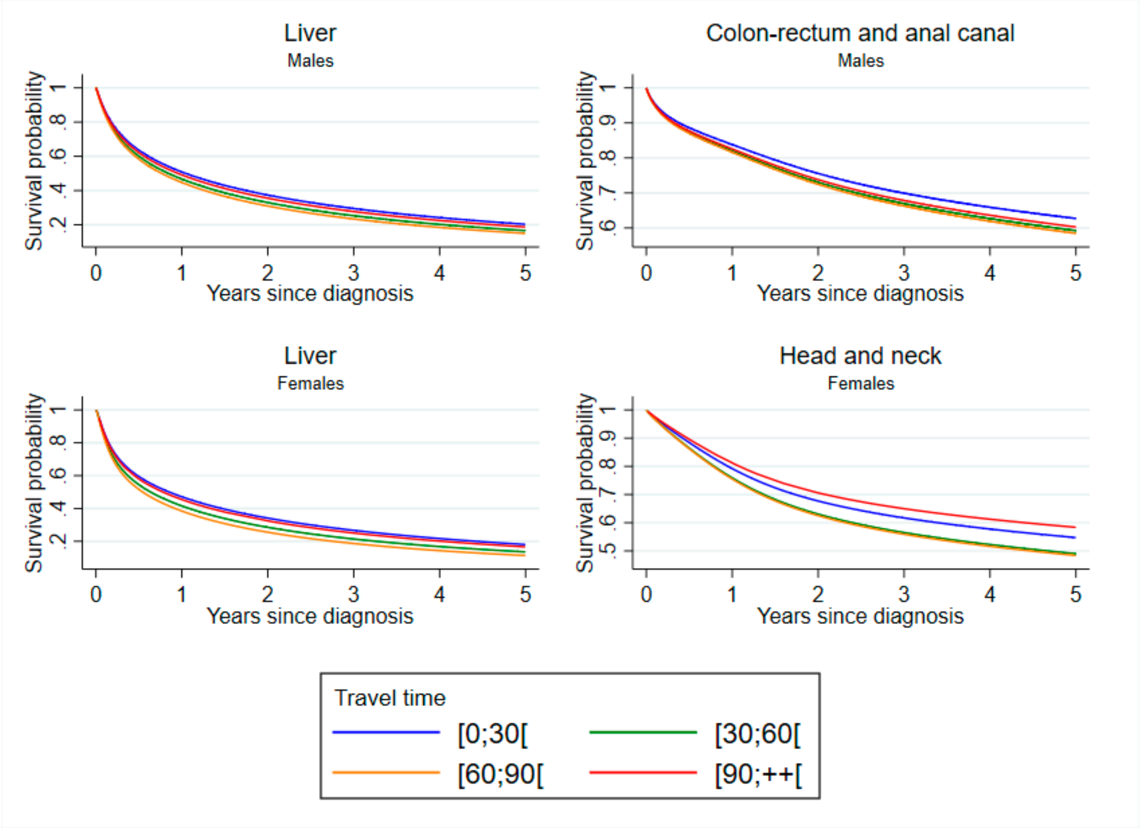

Travel time: travel time in minutes

Figure S3 – Survival probability – cases with no association

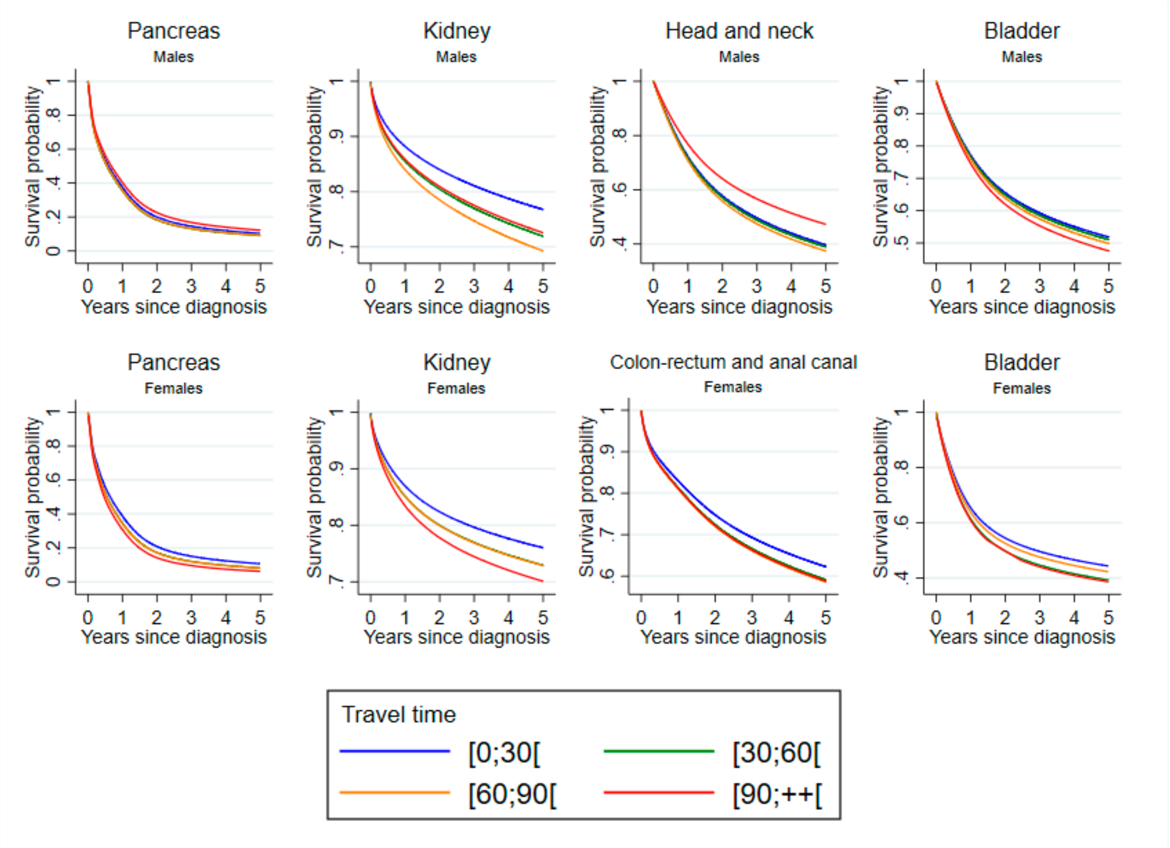

Travel time: travel time in minutes

Figure S4 – Survival probability – cases with better prognosis for remote patients

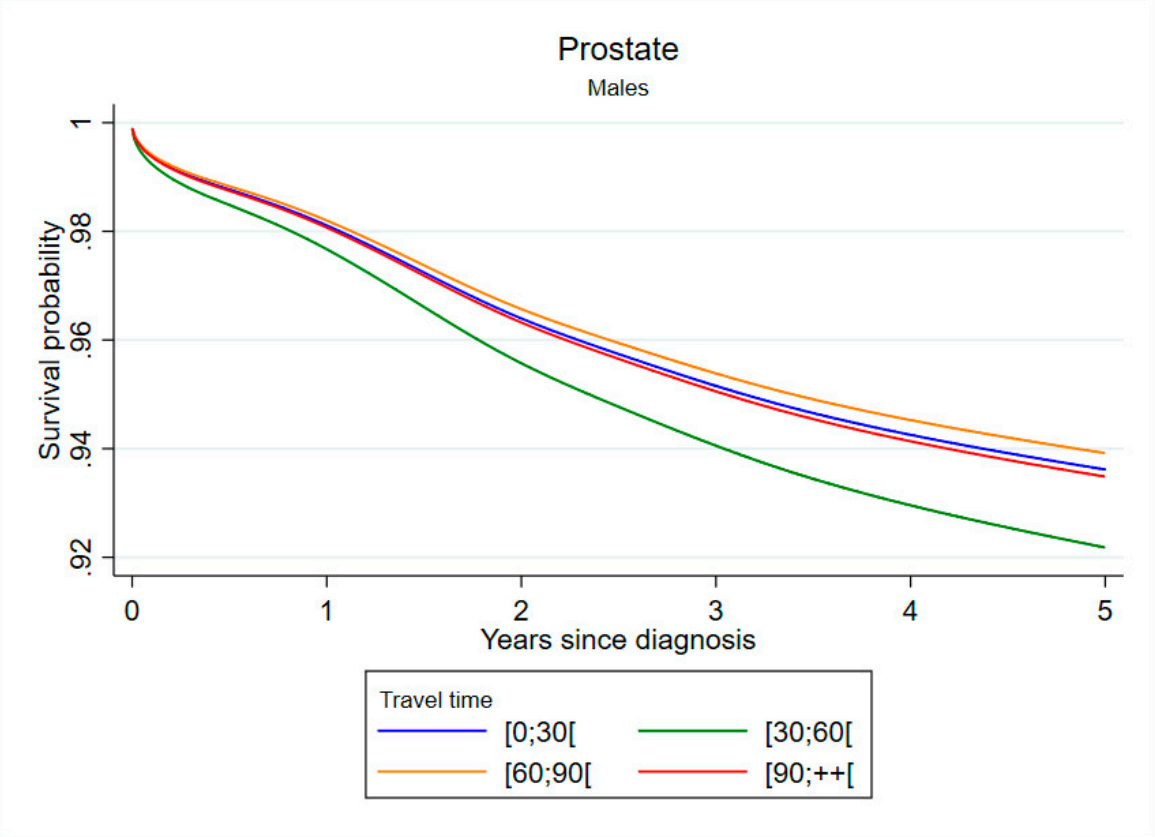

Travel time: travel time in minutes
